# 

**Published:** 1899-07-22

**Authors:** 


**Notices of Births, Deaths, and Marriages** are inserted in this column at a charge of 2s. 6d. for an announcement not exceeding four lines.

### NOTICE TO CORRESPONDENTS.

All MS., Letters, Books for Review, and other matters intended for the Editor should be addressed The Editor, "The Hospital," The Hospital Building, 28 & 29, Southampton Street, Strand, W.C.

The Editor cannot undertake to return rejected MS., even when accompanied by stamped directed envelope.

**Notice to Advertisers and Subscribers.**

All Advertisements, Orders for Copies of The Hospital, and Business Communications should be addressed to **THE MANAGER (Not to the Editor)**, The Hospital, The Hospital Building, 28 & 29, Southampton Street, Strand, London, W.C.

The Cost of Subscription, post free, is as follows:—Per week, 2½d.; per quarter, 2s. 8d.; per half-year, 5s. 3d.; per annum, 10s. 6d. Foreign:—Per annum, 15s. 2d., payable, in all cases, in advance.

**NOTICE.—Vol. XXV. of "The Hospital" (October, 1898, to March, 1899), in handsome cloth binding, is now on sale at the Office of this Journal, price 7s. 6d. Cases for binding the Numbers contained in this Volume 1s. 6d. Index to Vol. XXV., 2½d., post free.**

**The Monthly Part for August will be ready on August 1st. Price 6d., by post 8d.**

### BOOKS RECEIVED.

Scientific Press.
"The Principles of Mechanics." By Herbert Robson, B.Sc.,Lond.

Edward Arnold.
"Manual of Physiology." By Leonard Hill, M.B.

Whitley and Booth.
Annual Report. By D. Ainley, M.R.C.S., Medical Officer of Health for the County Borough of Halifax.

William Hodge, Glasgow.
Annual Reports. By John C. M'Vail, M.D., Medical Officer of Health for the Counties of Dumbarton and Stirling.

### Scraps and Gleanings.

Hospital Saturday for Gravesend this year will be on September 30th, and Hospital Sunday on October 8th.

The foundation-stone of the two memorial cottages given by Mrs. Fraser to the Victoria Homes, Guernsey, have been laid.

The report for 1898 of the Wimbledon Cottage Hospital shows that the income for 1898 fell short of that for the previous year by £76.

During the past year 5,814 out-patients have been treated at the Gravesend Hospital, 1,950 of which were casualties, and 937 dental cases.

A fancy fair is to be held at Portsmouth on September 20th, 21st, and 23rd, to raise funds for the furnishing of the new wards of the Royal Portsmouth Hospital.

Lord Rothschild, the President of the Royal Hospital for Diseases of the Chest, City Road, has contributed £100 towards the £1,500 required to enable the charity to claim a conditional donation of £500.

At the annual meeting of the Llanelly Hospital the secretary had the pleasant news to report of a balance on the right side of £200. The subscriptions of the working men of Llanelly during 1898 had been doubled.

One of the subscribers to the Bristol Royal Infirmary has offered to increase his subscription to £100 per annum and to guarantee such a sum for five years providing another £100 can be raised annually for five years either by increased or new subscriptions.

An urgent appeal is being made for help to assist in wiping out the debt of £543 which has accumulated during the last two years against the Memorial Hospital, Kendal, owing to insufficient subscriptions. This hospital has been in existence for nearly 29 years.

The enlargements and improvements at the Borough Fever Hospital, Rumworth, are now practically completed and will be shortly open for public use. By means of these extensions there will be room for 70 scarlet fever, 18 small-pox, and 16 typhoid fever patients.

The second sanatorium in connection with the consumption hospitals of Scotland at Bridge of Weir will be ready for occupation, it is hoped, by the end of this year. It is larger than the first house, containing, as it does, 40 beds. It is desired to be able to provide 200 beds at these homes, and a further sum of £35,000 is needed to enable this to be accomplished.

Owing to scarcity of funds the governors of the Southampton Eye, Ear, and Throat Hospital were obliged some time ago to abandon the treatment of ear and throat diseases. In consequence of this action a special department for the treatment of these diseases is to be formed at the Royal South Hants Infirmary and a surgeon appointed to take charge of the same.

On the occasion of the second anniversary of the opening of the Victorian Convalescent Home, Bognor, the Mayor of Guildford informed the committee that they were to receive a handsome sum from the recent Hospital Saturday Fund collection; and the Mayor of Richmond informed the President that he would be ready to contribute the last £100 to complete the £10,000, which it was hoped would soon be collected.

A pleasing announcement was made at the recent monthly meeting of the committee of management of the Victoria Hospital for Nervous Diseases, Belfast, namely, that the satisfactory state of the hospital's finances warranted the acceptance of estimates for the complete equipment of an internal department, the arrangements for which are to be immediately put in hand.

Among the hospitals most recently fitted with efficient means of protection in case of fire is the Hospital for Sick Children, Great Ormond Street, W.C., which is now in possession of three Merryweather chute fire escapes, affording rapid and safe means of egress from a building in case of an emergency. The Middlesex Hospital has also just been supplied with six "London Brigade" Merryweather hand pumps.

The Earl and Countess of Jersey have generously invited 600 boys and girls of the National Refuges for Homeless and Destitute Children, including the sailor boys of the "Arethusa" and "Chichester" training ships, Greenhithe, Kent, to spend the day at Osterley, Isleworth, on the 19th instant. Boys from the Bisley Farm Schools and 200 girls from the society's homes at Sudbury and Ealing will share in the festivities.

### The Student of Medicine.

### APPOINTMENTS.

G. A. Barrow, M.R.C.S., L.R.C.P., appointed Assistant Medical Officer to the Manchester Hospital for Consumption and Diseases of the Throat and Chest. F. C. Brodie, M.B., B S., appointed Admiralty Surgeon and Agent at Sandoun. P. Foster, M R.C.S., L.R.C.P.Lond., appointed Assistant Resident Medical Officer to the Royal National Hospital for Consumption, Ventnor, I.W. H. Haigh, M.A.Cantab., M.R.C.S , L.R.C.P., appointed Medical Officer to the Convalescent Home, Meltham Mills, near Huddersfield. A. J. Hall, B.A., M.B.Cantab., M.R.C.P., appointed Professor of Pathology in University College, Sheffield. H. E. Harris, B.A., M.B.Cantab., F.R.C S.Eng., L.R.C.P.Lond., appointed Surgeon to Out-patients to the Bristol Royal Hospital for Sick Children and Women. J. Horne, M.B., B.C.Cantab., appointed Honorary Surgeon to the Metropolitan Ear, Nose, and Throat Hospital. R. Lake, F.R.C.S.Eng., appointed Honorary Surgeon to the Metropolitan Ear, Nose, and Throat Hospital. C. Lewis, M.D., appointed House Physician to the General Hospital, Birmingham. G. McKellar, M.D., C.M., D.P.H., appointed temporarily an Assistant Surgeon to the British Ophthalmic Hospital, Jerusalem. J. H. Marsh, M.R.C.S.Eng., L.R.C.P.Lond., appointed Medical Officer of Health for the Borough of Macclesfield. A. W. Quait, M.R.C.S Eng., L.R C.P. Lond., appointed Medical Officer of the Mundesley District of the Erpingham Union. H. Skelding, M.B., B.C.Cantab., M.R C.S., L.S.A., appointed Assistant Surgeon to the Bedford County Hospital.

